# Dietary Modulation of *Drosophila* Sleep-Wake Behaviour

**DOI:** 10.1371/journal.pone.0012062

**Published:** 2010-08-10

**Authors:** James H. Catterson, Seymour Knowles-Barley, Katherine James, Margarete M. S. Heck, Anthony J. Harmar, Paul S. Hartley

**Affiliations:** 1 Centre for Cardiovascular Science, Queen's Medical Research Institute, The University of Edinburgh, Edinburgh, Scotland; 2 School of Informatics, The University of Edinburgh, Edinburgh, Scotland; Centre National de la Recherche Scientifique, France

## Abstract

**Background:**

A complex relationship exists between diet and sleep but despite its impact on human health, this relationship remains uncharacterized and poorly understood. *Drosophila melanogaster* is an important model for the study of metabolism and behaviour, however the effect of diet upon *Drosophila* sleep remains largely unaddressed.

**Methodology/Principal Findings:**

Using automated behavioural monitoring, a capillary feeding assay and pharmacological treatments, we examined the effect of dietary yeast and sucrose upon *Drosophila* sleep-wake behaviour for three consecutive days. We found that dietary yeast deconsolidated the sleep-wake behaviour of flies by promoting arousal from sleep in males and shortening periods of locomotor activity in females. We also demonstrate that arousal from nocturnal sleep exhibits a significant ultradian rhythmicity with a periodicity of 85 minutes. Increasing the dietary sucrose concentration from 5% to 35% had no effect on total sucrose ingestion per day nor any affect on arousal, however it did lengthen the time that males and females remained active. Higher dietary sucrose led to reduced total sleep by male but not female flies. Locomotor activity was reduced by feeding flies Metformin, a drug that inhibits oxidative phosphorylation, however Metformin did not affect any aspects of sleep.

**Conclusions:**

We conclude that arousal from sleep is under ultradian control and regulated in a sex-dependent manner by dietary yeast and that dietary sucrose regulates the length of time that flies sustain periods of wakefulness. These findings highlight *Drosophila* as an important model with which to understand how diet impacts upon sleep and wakefulness in mammals and humans.

## Introduction

Although the evolutionary significance of sleep is not understood, it contributes to the viability, longevity, health and cognitive abilities of a wide range of organisms, from invertebrates to humans. Many reports associate the disruption of sleep in mammals and humans with the development of metabolic syndrome, type 2 diabetes and increased risk of cardiovascular disease [1], [2], [3], [4], [5], [6], yet the cause and effect relationship between sleep disturbances and disease remains largely unaddressed. The fruit fly *Drosophila melanogaster* is an important model with which to analyse both sleep and metabolism in adult organisms, however little information regarding the effect of diet and metabolism upon fly sleep is available.

*Drosophila* and mammalian sleep exhibit important similarities, such as the requirement for dopaminergic and GABAergic signaling, the existence of an intrinsic (circadian) timing mechanism, the impact of homeostatic (‘tiredness’) factors and sustained arousal in response to methamphetamine, caffeine and modafinil [7], [8], [9], [10], [11], [12], [13]. In addition, flies and mammals exhibit similar patterns of neurological activity when in different states of arousal [14], [15]. Although the effect of diet upon feeding, foraging, reproduction and longevity has been studied for many years [16], [17], [18], [19], a detailed study of how diet affects *Drosophila* sleep-wake behaviour has not been performed.

In our current work, we aimed to establish how diet affects the architecture of *Drosophila* sleep-wake behaviour. Using automated behavioural monitoring, we find that in male flies, dietary yeast promotes arousal from nocturnal sleep bouts. In contrast, dietary carbohydrate determines how long males and females sustain activity when awake. We also demonstrate that arousal from nocturnal sleep exhibits an ultradian rhythm with a periodicity of 85 minutes. We conclude that diet profoundly influences the architecture of *Drosophila* sleep-wake behaviour and discuss the relevance of these finding to mammalian sleep.

## Methods

### Reagents

All stock chemicals and agarose, sucrose, yeast extract, Metformin and 3-iodo tyrosine were from Sigma (Dorset, UK).

### Fly lines and maintenance

The *w^1118^* and *Canton S Drosophila* lines were obtained from the Bloomington Stock Centre. Flies were propagated on a maize-yeast diet, prepared as follows: 14L of H_2_O, 150g agar, 1100 g sucrose, 620 g brewers yeast, 1000 g maize, 80 g dried live yeast 45 ml propionic acid and 38 g nipagin mixed with 380 ml ethanol and maintained at 25°C in a humidified incubator on a 12 hr∶12 hr light∶dark cycle.

### Monitoring of sleep-wake behaviour

Three to six day old male or female flies that had been socially housed (see [20]) were anaesthetised using CO_2_ and single flies transferred to a 5 mm ×65 mm polycarbonate tube containing food (see below). The tube was sealed at the food end with Parafilm (Pechiney Plastic Packaging Company, IL, USA), with the opposing end sealed with a cotton wool plug to allow for air transfer. Fly sleep-wake behaviour was monitored using the Drosophila Activity Monitoring System (DAMS, TriKinetics, Waltham, MA, USA). All experimental procedures were carried out at 25°C in a humidified incubator. Flies were monitored for at least four days on a 12 hr∶12 hr light∶dark cycle. All analysis was done with data collected from days 2 to 4 after flies were placed into the DAMS. Sleep was regarded as a period of five minutes without beam crossing (as defined previously by other investigators [8], [9]). Data was collected using TriKinetics software and analysed in Excel (Microsoft, Redmond, WA, USA ) and Clocklab (Actimetrics, IL, USA).

### Modulation of diet

Flies in DAMS tubes (or 7 ml bijou bottles for biochemical assays) were provided with the following diets: agar-sucrose (AS, 1.37% agar, 5% sucrose) or agar-sucrose-yeast (ASY, the AS diet with 2% yeast extract). In some experiments the sucrose concentration of the AS diet was increased to 35%. Drugs were added to the diets to a final concentration of 5 mg/ml for 3IY, and 1 mM or 10 mM for Metformin.

### Provocation of arousal

Flies within DAMS were provoked into activity by swiftly dragging an empty DAMS tube twice (in quick succession) across the DAMS array at ZT16 (four hours after lights off). Preliminary studies confirmed that this provocation aroused approximately half of the male flies within the DAMS tube when on the AS diet and is similar to the mechanical stimulation of flies used to assess arousal thresholds used in other studies [9].

### Capillary Feeder Assay (CAFE Assay)

A 7 mL bijou vial filled with 1 ml of (1%) agar, to ensure humid conditions, sealed with Parafilm (Alpha Laboratories Ltd, Hampshire, UK). Four holes in the Parafilm that were equally spaced apart, were made using a 26-gauge needle to ensure adequate air circulation. Through the Parafilm was inserted a truncated 200 µl pipette tip which held a graduated 5 µl disposable glass capillary tube (Camag, Muttenz, Switzerland) containing liquid food (as described in the text) supplemented with blue food dye (Langdale, Market Harborough, UK) to aid measurement of feeding. For all experiments, a mineral oil overlay (0.1 µl) was used to minimize evaporation. Food ingestion was measured every 24-hr for five consecutive days. Each experiment included an identical, CAFE chamber without flies to determine evaporative losses (typically 10% of ingested volumes), which were subtracted from experimental readings [21].

### ATP Assay

The ATP concentration of flies was determined using the Roche ATP Bioluminescence Assay Kit HS II (Roche, West Sussex, UK). Briefly, flies were frozen at −80°C for 10 minutes and homogenised under ice-cold lysis buffer (provided in kit) for 1 minute using a Kontes pellet pestle (Kontes Glass Company, NJ, USA). Homogenates were centrifuged at 13,000 rpm in a bench-top microcentrifuge, incubated with luciferin substrate and bioluminescence determined using a Glomax Multi+ detection System (Promega, Hampshire, UK).

### Analysis of Ultradian Rhythms

For the analysis of ultradian rhythms in arousal the data for three nights locomotor activity monitoring for individual flies was combined into one 24 h period and the probability of any fly ending a period of inactivity at each time point was calculated and weighted by the length of the preceding inactivity. A moving average of 30 minutes was applied to smooth the data. This produced a weighted wake time preference for the population of flies. Chi-squared periodogram analysis was used to quantify the ultradian rhythm [22]. This method provides a significance test based on the chi-squared distribution and remains accurate under the presence of noise [23]. Linear regression was used to fit a straight line to weighted wake time preference data and the regression fit was subtracted to remove any linear trend. Periodogram analysis was then applied to the data for periods between 10 and 270 minutes.

### Statistics

One-way analysis of variance (ANOVA) followed by Tukey's post-hoc test was used to identify differences between three or more means derived from uneven sample sizes. The student's unpaired *t*-test was used to identify significant differences between two means of uneven sample size. A statistical difference of P<0.05 was regarded as significant.

## Results

### Dietary yeast affects *Drosophila* sleep in a sex-dependent manner

The study of *Drosophila* sleep-wake behaviour has typically been performed with flies fed an agar-based diet that contains 5% sucrose. Whilst this diet is sufficient for fly viability it is devoid of macro and micronutrients that might be expected to influence sleep-wake behaviour. We therefore tested the hypothesis that dietary yeast extract (2% in agar supplemented with 5% sucrose) may promote arousal in flies.

**Figure 1:**
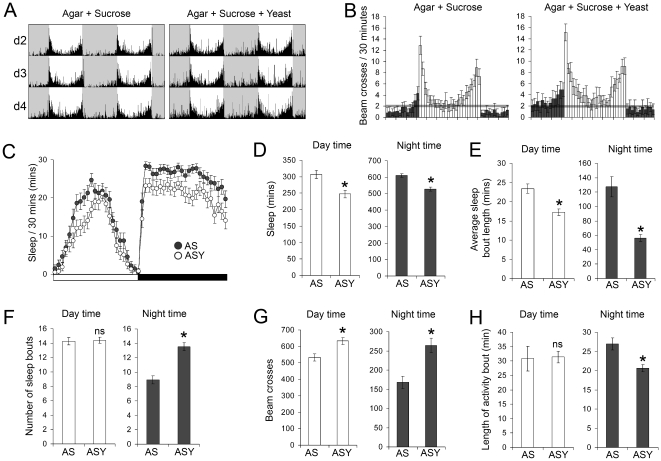
Effects of dietary yeast extract on sleep-wake behaviour of male *Drosophila*. Male *w^1118^* flies were housed in polycarbonate tubes and provided with agar containing 5% sucrose (AS) or agar, 5% sucrose and 2% yeast extract (ASY). Locomotor activity was recorded as the number of times a fly broke the path of an infra-red beam at the midpoint of the tube. Periods of 5 minutes without beam crossing were regarded as a single period of sleep. (**A**) Actograms showing averaged beam crossing data for three consecutive days (d2, d3 and d4). The light and shaded areas denote the 12-hour periods of light and darkness of the 24-hour cycle. A single day's data is re-plotted on the following line so that the relationship between the light-dark cycle and the rhythm of locomotor activity can be seen better. (**B**) Averaged beam crossing data binned to every half-hour of the 24-hr cycle for flies fed the different diets; dark bars represent night and day activity, respectively. The grey bar denotes an average of 2 beam crosses per half hour and is presented to aid comparisons of the data. (**C**) Minutes of sleep per 30 minutes. The white and black bar represents the 12 hour light and dark phases of the 24 hour cycle. (**D**) Total amount of time spent asleep for flies fed the agar-sucrose (AS) and agar-sucrose-yeast (ASY) diets. (**E**) The average length of each sleep bout by flies fed the different diets. (**F**) The mean number of sleep bouts. (**G**) The amount of locomotor activity undertaken by flies on the different diets. (**H**) Average length of activity bouts. *P<0.01; #P<0.05; n = 32 flies for each diet in A, B and C; rest of data is n = 100 flies for each diet, data was pooled from five independent trials.

Male *w^1118^* flies provided either with 5% sucrose (AS) or the AS diet containing 2% yeast extract (ASY), exhibited a normal bimodal pattern of behaviour with distinct periods activity approximating the time of lights on and lights off (Figure 1 A & B). However, flies provided with the ASY diet exhibited a reduction in both daytime and nighttime sleep compared to flies on the AS diet (Fig. 1C & D), that was associated with shorter, more numerous nocturnal sleep bouts (Figs. 1E & F), and increased locomotor activity (Fig. 1G). Although the ASY diet had no effect on the length of daytime activity bouts, it caused a small but significant shortening of nocturnal activity bout length (Fig. 1H). Similar findings were obtained when male flies of the wild type *Canton S* line were used (more nocturnal locomotor activity, reduced nocturnal sleep and shorter, more frequent nocturnal sleep bouts; data not shown).

**Figure 2:**
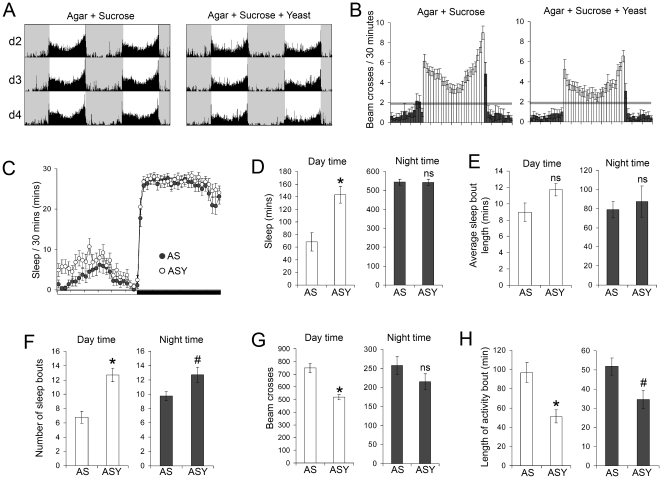
Effects of dietary yeast extract on sleep-wake behaviour of female *Drosophila*. (Legend as for figure 1). *P<0.01; #P<0.05; n = 48 flies for each diet, data pooled from two independent trials.

The females' response to the ASY diet was characterized by a notable decrease in daytime locomotor activity, a finding that was in stark contrast to the behaviour of males (Fig. 2A, B & G). This change in behaviour was not associated with an alteration to the amount of nocturnal sleep or locomotor activity (Fig. 2C, D & G), nor any change to the length of either daytime or nocturnal sleep bout length (Fig. 2E). However (and similar to the behaviour of male flies), the ASY diet led to more bouts of sleep (Fig. 2F), due to a shortening of daytime and nocturnal activity bouts (Fig. 2H). Again, similar findings were obtained for female Canton S flies (a significant reduction in daytime locomotor activity on the ASY diet and no change to total nocturnal sleep or nocturnal sleep bout length; data not shown). Providing flies with a source of yeast did not affect food intake by either sex (Figure S1), thus these results are due to the ingestion of yeast rather than a change to sucrose intake.

### Dietary yeast promotes arousal in males

**Figure 3:**
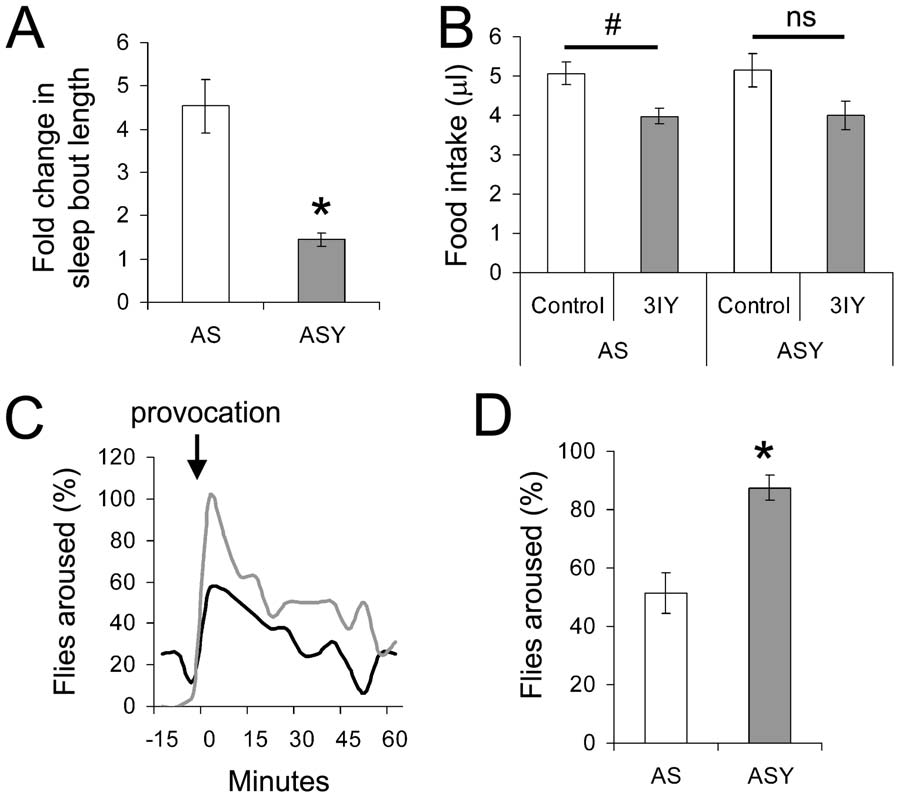
Dietary yeast affects arousal. The behaviour of male flies was assessed as described in Figure 1 and the Methods section. (**A**) The graph shows the fold change in sleep promoted by 5 mg/ml 3IY, an inhibitor of dopamine synthesis, when supplemented in either an agar-sucrose (AS) or yeast-containing AS diet (ASY). (**B**) CAFE data showing that amount of food ingested after five days on the AS and ASY diets, supplemented with the dopamine synthesis inhibitor, 3IY. (**C**) Average percentage of flies fed the AS diet or the ASY diet (black and grey line, respectively) aroused in response to a mechanical provocation (arrow) performed at ZT16. (**D**) Quantified data for the provocation test. #P<0.01, *P<0.01; CAFE assays used 9–10 flies per treatment; for the arousal provocation tests (C and D) two independent trials were performed involving 16 flies for each treatment.

Arousal from sleep in *Drosophila* is controlled by dopaminergic neurons [13], [24]. Changes to the mechanism controlling arousal manifest as a shortening or lengthening of each sleep bout [25]. To verify that dietary yeast promoted arousal in males, we tested the hypothesis that the ASY diet may limit the sleep-promoting effect of 3-iodo tyrosine, a drug that inhibits dopamine synthesis [26]. Consistent with this hypothesis, sleep bouts were approximately 4.5 times longer when 3IY was added to the AS diet, but only marginally increased when added to the ASY diet, confirming that the ASY diet modulated arousal (P<0.01; Fig. 3A). This change could not be accounted for by alterations to food intake by 3IY, which was reduced to the same extent on the AS and ASY diets (Fig. 3B). Further verification that dietary yeast promoted arousal in males, was obtained by provoking flies into activity during the nocturnal period (ZT 16, four hours after lights off, Fig. 3C). The percentage of flies that became active on the AS diet was 56% and 31% (trials one and two), whereas 100% and 81% of the flies on the ASY diet were aroused by the provocation (P<0.01; Figure 3D).

### Dietary yeast does not affect an ultradian rhythm of arousal in male flies

**Figure 4:**
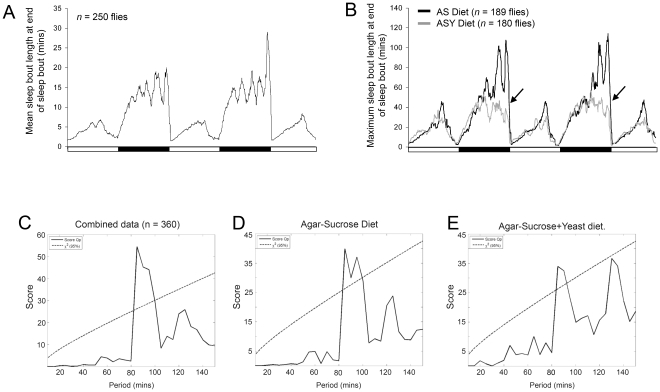
Arousal from sleep is ultradian and disrupted by dietary yeast. The time of day that a male fly aroused from a sleep bout was recorded and the length of that sleep bout was calculated and plotted as a 1-hour running average over 2.5 consecutive days. (**A**) Data from 250 different male flies from five independent experiments is presented. During the daytime there is no obvious ultradian rhythm of arousal, whereas at night there are several discrete epochs during which flies are more or less likely to arouse from sleep. (**B**) The effect of the AS (black line) and ASY (gray line) diets upon the ultradian pattern of nocturnal arousal. Data for the maximum length of sleep bouts is presented. Flies fed the AS diet show an ultradian rhythm of arousal, whereas, flies on the ASY diet have similar periodicity ultradian rhythm of arousal but lower peaks of sleep length due to shorter periods of sleep on the ASY diet (arrows). (**C**) Periodogram analysis of the data combined from all experiments (n = 360 male *w^1118^* flies) established that arousal occurs according to a significant ultradian rhythm with a periodicity of 90 minutes. (**D**) Periodogram showing a significant ultradian rhythm of nocturnal arousal for male flies fed sucrose only. (**E**) The periodogram for male flies fed sucrose and yeast extract shows a significant ultradian rhythm of 85 minutes and a trend towards a longer frequency rhythm, of approximately 130 minutes, that does not reach significance. The black and white bar denotes the 12-hour light and dark periods. n = 189 and 180 flies from independent experiments for the AS and ASY diets, respectively.

Although an individual fly can sleep continuously for several hours and even throughout the entire night, the averaged data from a cohort of experimental flies consistently indicated that arousal from nocturnal sleep exhibited an ultradian rhythmicity (Fig. 4). Although dietary yeast shortened nocturnal sleep bouts (see Fig. 1E and Fig. 4B), flies on both the AS and ASY diets show a peak rhythmicity at 85 minutes, significant at a 95% confidence level (Fig. 4C–E). A second ultradian rhythm of approximately 130 minutes was also evident and which neared statistical significance in flies fed the ASY diet (compare Fig. 4D with 4E). This ultradian rhythm of arousal from nocturnal sleep was also observed in female *w^1118^* flies and in *Canton S* flies (not shown).

### Effect of dietary sucrose concentration on sleep-wake behaviour

**Figure 5:**
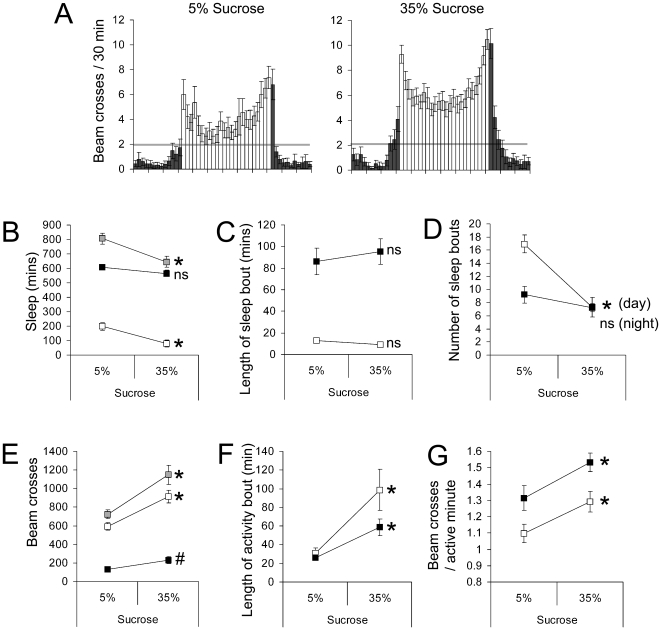
Effect of dietary sucrose on male *Drosophila* sleep-wake behaviour. Male *w^1118^* flies were fed diets containing different sucrose concentrations and their sleep-wake behaviour monitored for three consecutive days. (**A**) Activity plots binned to every half-hour of the 24-hr cycle for flies fed the different diets; dark bars represent night and day activity, respectively. The grey bar denotes an average of 2 beam crosses per half hour and is presented to aid comparisons of the data. (**B**) Sleep (periods of 5 minutes without a fly crossing the beam) of flies on the two diets. (**C**) Length of sleep bouts. (**D**) The number of sleep bouts. (**E**) The amount of locomotor activity undertaken by flies on the different diets. (**F**) Average length of activity bouts. (**G**) Intensity of activity bouts. Grey boxes refer to 24 hr data, open boxes refer to the 12 hour light period and filled boxes relate to data collected during the 12-hour dark period. *P<0.01; #P<0.05; n = 32 flies for each diet and is representative of four independent trials.

**Figure 6:**
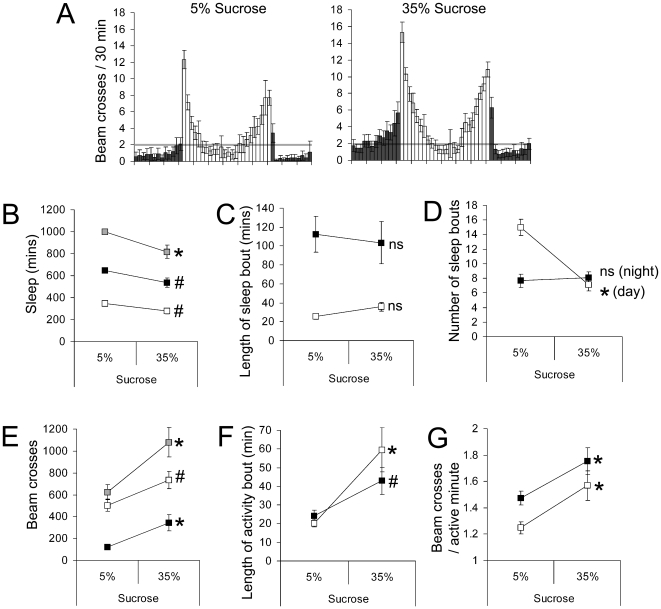
Effect of dietary sucrose on female *Drosophila* sleep-wake behaviour. Female *w^1118^* flies were fed diets containing different sucrose concentrations and their sleep-wake behaviour monitored for three consecutive days. (**A**)–(**G**) Legend as for figure 5. *P<0.01; #P<0.05; n = 15–16 flies for each diet and is representative of two independent trials.

We next analysed the effect of dietary sucrose on sleep-wake behaviour. Although locomotor activity and flight are known to be affected by dietary carbohydrate (see [27]), the effect of carbohydrate on sleep has not been addressed. We found that increasing the sucrose content of the AS diet from 5% to 35% led to a significant increase in locomotor activity that was associated with reduced total sleep in both males and females (Figs. 5 & 6, respectively). The increase in locomotor activity was due (in both sexes) to longer bouts of activity and an increase in the intensity of locomotor activity (i.e. more beam crossing per waking minute). Despite these changes to locomotor activity, there was no change to the length of daytime or nocturnal sleep bouts, indicating that changes to the concentration of dietary sucrose were not sufficient to alter the arousal threshold and that the loss of sleep on the higher sucrose concentration was due to flies maintaining longer periods of activity when awake. We also confirmed that flies ingested the same amount of sucrose on the low and high sucrose diets (Figure S2), indicating that the effects of dietary sucrose were related to the amount of sucrose in each ‘meal’ rather than a change to the total amount of sucrose ingested over the day. A similar increase in both total daytime locomotor activity and daytime activity bout length was recorded for male and female wild type (*Canton S*) flies (data not shown).

### Metabolic inhibitors affect locomotor activity but not sleep

**Figure 7:**
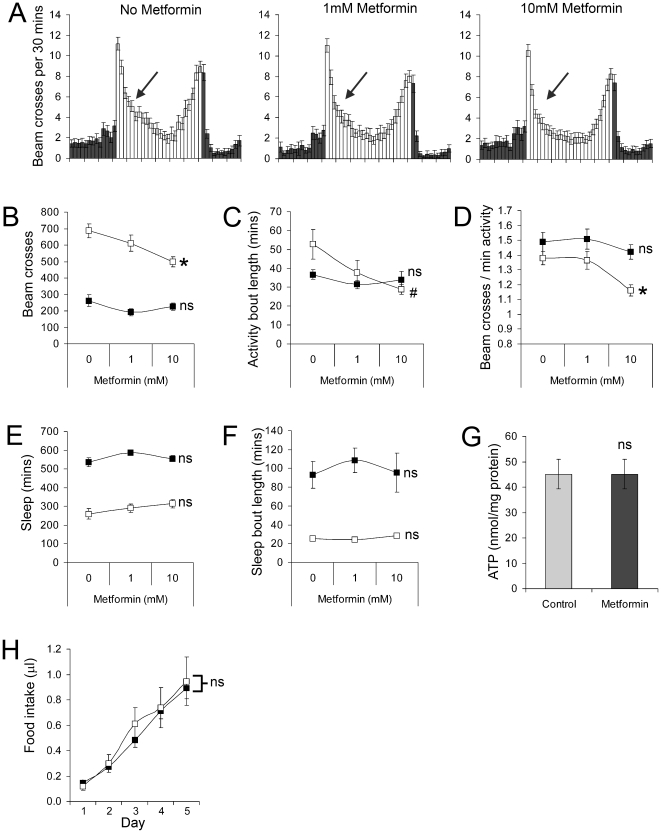
The effect of Metformin on sleep-wake behaviour, basal ATP and food intake. (**A**) Flies were provided with agar based diet containing 30% sucrose, supplemented with Metformin at the stated dose. The three charts show mean (±SEM) beam crosses from three days of behavioural monitoring. The arrows are identically placed within each chart and point to the decrease in morning activity. (**B**) Locomotor activity (beam crosses) on diets containing difference concentrations of Metformin. (**C**) Length of each activity bout. (**D**) Intensity of locomotor activity. (**E**) Total amount of sleep. (**F**) Length of each sleep bout. (**G**) Basal ATP in flies. (**H**) Food intake by flies on control (filled boxes) or 10 mM Metformin (open boxes), as measured by CAFE assay. In B–F open and filled boxes relate to data collected during the day and night of the 12 hr∶12 hr light-dark cycle, respectively. Data are expressed as the mean (±SEM) and is pooled from two impendent trials using a total of 32 flies for each treatment. *P<0.01, # = P<0.05, ns  =  not significant.

The data from several different experiments and trials overwhelmingly indicated that increases in locomotor activity did not result in compensatory increases to total sleep time nor changes to arousal status. Hence the amount of time a fly sleeps and the ‘depth’ of sleep is uncoupled from prior amounts of activity, a conclusion that agrees with the findings from sleep-deprivation studies [8]. This predicts that decreased locomotor activity will not reduce the amount of sleep nor would it affect sleep bout length. To address this question we fed male flies Metformin, a drug which inhibits complex one of the respiratory chain [28], and which would be predicted to reduce locomotor activity due to limiting ATP production in muscle. Consistent with this we found that Metformin significantly reduced the locomotor activity of flies by shortening the length and intensity of daytime (but not nighttime) activity bouts (Fig. 7A–D). Importantly, this reduction to daytime activity occurred in the absence of any change to the total amount of daytime or nocturnal sleep (Fig. 7E) and the length of daytime or nocturnal sleep bouts (Fig. 7F). Interestingly, we recorded no significant change to basal ATP levels in flies fed Metformin (Fig. 7G), nor any change to the amount of sucrose ingested (Fig. 7H), suggesting that energy homeostasis may have been maintained by the reduction in locomotor activity. These findings further support the conclusion that total sleep time and the arousal threshold are uncoupled from the amount of locomotor activity and energy expenditure in *Drosophila*.

## Discussion

*Drosophila melanogaster* is an important model organism with which to study the relationship between diet and behaviour. In the past decade *Drosophila* has been used to study sleep, a behaviour that impacts upon the physiology and viability of humans and a phylogentically diverse array of animal models. A significant limitation to the understanding of sleep and its impact upon health, is the lack of inexpensive, tractable models and a high degree of variability in sleeping behaviours across phyla. The importance of understanding the physiological relevance of sleep is highlighted by recent research into obesity, which has linked sleep disruption with the development of metabolic syndrome, type II diabetes and cardiovascular disease [1], [2], [3], [4], [5], [6]. Thus, the aim of our current study was to characterise the effects of diet on *Drosophila* sleep-wake behaviour. We present data that demonstrates for the first time that dietary yeast, a food eaten by flies in the wild and under laboratory conditions, fragments sleep-wake behaviour by promoting arousal in males and by shortening periods of locomotor activity in females. We also demonstrate that *Drosophila* can exhibit an ultradian pattern of arousal from sleep, a finding of considerable interest, as it resembles the pattern of sleep in mammals and humans. Finally we show that dietary carbohydrate concentration determines the length of time that male and female flies sustain periods of wakefulness but that, on its own, it has no effect on arousal.

Sleep exhibits a highly structured, ultradian architecture in humans and rodents that is characterised by several hours of non rapid eye movement (NREM) sleep during the early part of the night, followed by shorter periods of rapid eye movement (REM), interspersed by NREM until arousal. Our observation that flies also show an ultradian pattern of arousal from nocturnal sleep is further evidence that the mechanisms controlling sleep in flies may be conserved in mammals and humans. Although ultradian locomotor activity is reported for flies without functional circadian oscillators [29], [30], our data are the first to show ultradian rhythmicity of arousal in flies with an intact circadian oscillator. We found that flies of both sexes aroused during several discrete epochs during the night. Quantitative analysis of males revealed that the ultradian rhythm's periodicity was 85 minutes on both the AS and ASY diets, but that there is a trend towards a second, longer rhythm of approximately 130 minutes when flies are fed yeast extract. It is important to note that the expression of ultradian behaviour was variable and that statistical significance was only reached when the analysis was performed on a considerable number of flies. Nonetheless, these findings indicate the existence of an ultradian oscillator within the fly brain that controls the initiation of arousal from nocturnal sleep. The mechanism controlling this ultradian rhythmicity of arousal remains unclear. Several reports indicate that dopamine regulates arousal and sleep in flies [13], [24], suggesting the ultradian rhythm of nocturnal arousal in *Drosophila* may reflect the ultradian regulation of dopamine know to exist in mammals [31], [32].

Studies of *Drosophila* sleep do not currently utilise a standard diet. Instead, diets range from the medium used for fly propagation (typically an agar based diet containing high concentrations of carbohydrate and yeast), to a nutritionally-limited agar based diet that contains sucrose but which is devoid of other nutrients. Our data indicate that dietary yeast reduces the arousal threshold in males and shortens the length of wakeful periods in females, leading to a more fragmented sleep-wake architecture for both sexes. The relevance of these adaptations is unclear, however diets that promote nocturnal activity may be advantageous for the reproductive strategy of males, which have increased sex drive at night [33] and court females during the day and night [34], [35]. Whilst the omission of dietary yeast may be of little consequence within a given study of fly sleep, our findings highlight an important source of variation between different experimental protocols.

How dietary yeast affects the arousal behaviour of males is not clear. Dietary yeast yields amino acids such as glutamic acid, L-tryptophan, and L-tyrosine for the synthesis of γ-butyric acid, serotonin, octopamine and dopamine; salts such as potassium, and cholesterol - a precursor or 20-hydroxyecdysone, all of which affect sleep in flies [7], [36], [37], [38], [39]. In rodents and mammals the ratio of carbohydrate to protein, and the ratio of amino acid species within a meal, affect both the concentration of amino acid species within the blood and the rate at which neuroactive monoamines are synthesized [40], [41]. Similarly, the regulation of electrolytes in *Drosophila* by the Malpighian tubules is highly dependent upon the concentration of amino acids within the hemolymph [42]. Therefore, a simple, linear correlation between dietary constituents and the amounts and quality of sleep or wakefulness is unlikely to exist. In stark contrast to the effect on male behaviour, yeast reduced daytime activity and increased daytime sleep in females, a finding similar to that reported by Broughton *et al*., [43]. As dietary yeast did not affect sleep bout length in females, we conclude that the effect of dietary yeast on the females' daytime sleep is due to the shortening of activity bouts and unrelated to changes in arousal. This sex-dependent difference in the response to dietary yeast is not understood but may involve insulin signaling, which is implicated in the regulation of sexually dimorphic locomotor behaviour and the regulation of sleep [44], [45], [46], [47].

In contrast to yeast, we found that dietary carbohydrate (in the form of sucrose) regulated the length of time that flies remained active whist having no influence on the length of sleep bouts. Thus, the period of sleep immediately following a bout of locomotor activity is not related to the amount of exercise undertaken during that bout: this uncoupling of sleep from ‘exercise’ is highlighted by the effect of Metformin, an inhibitor of oxidative phosphorylation, that caused reduced locomotor activity but which had no effect on any parameter related to sleep or arousal. The restriction of Metformin's effect to the morning is consistent with a diurnal influence on food intake (and therefore drug ingestion), which is maximal at this time [48]. Models of sleep deprivation have demonstrated that the amount of sleep following deprivation does not correlate with the amount of locomotor activity during deprivation [8]. Similarly, others have reported either no, or only a weak correlation between waking activity (activity per waking minute) and sleep [49], [50]. Therefore, it appears that fly sleep does not contribute significantly to metabolic homeostasis, which is far better maintained by adaptations to food intake and locomotor activity. However, this conclusion does not exclude the possibility that pathological and/or chronic disruption of oxidative phosphorylation or glucose metabolism, may impact upon sleep by modulating the function of neurons controlling arousal.

### Conclusions

Diet has profound, sex-dependent effects on the sleep architecture of *Drosophila*. Flies exhibit an ultradian rhythm of arousal that resembles the cyclical sleep patterns of mammals and humans. Dietary yeast promotes the fragmentation of sleep-wake behaviour in both sexes but by different mechanisms: in males it reduces the arousal threshold and thus shortens bouts of sleep; whereas in females it shortens bouts of locomotor activity. When flies of either sex awaken from sleep, the length of time they remain awake and the amount of activity they undertake, is dependent upon the sucrose content of their diet. Changes to total locomotor activity do not correlate with changes to the amount of sleep, suggesting that sleep is uncoupled form energy expenditure in *Drosophila*. These findings indicate that *Drosophila* will be a valuable model with which to understand the relationship between diet, sleep and physiology that exists in mammals and humans.

## Supporting Information

Figure S1 Dietary yeast does not affect food intake. CAFE assays were used to monitor food intake by male and female flies for five days. The provision of 2% yeast extract in the 5% sucrose-water had no effect on food intake. ns  =  not significant; n = 8–10 flies per diet.
(0.08 MB TIF)

Figure S2 Sucrose ingestion on diets containing different sucrose concentrations. Sucrose ingestion was monitored by CAFE assay for five days. The provision of sucrose at either 5% or 35% in water, had no effect on the total amount of sucrose ingested over a five day period. (n = 8–10 male flies per treatment; P>0.05 by two way ANOVA).
(0.08 MB TIF)
