# Supplementary figures and images for: Dietary Modulation of Drosophila Sleep-Wake Behaviour
Source: PLoS One. 2010 Aug 10;5(8):e12062. doi: 10.1371/journal.pone.0012062 (PMC2919389; doi:10.1371/journal.pone.0012062)

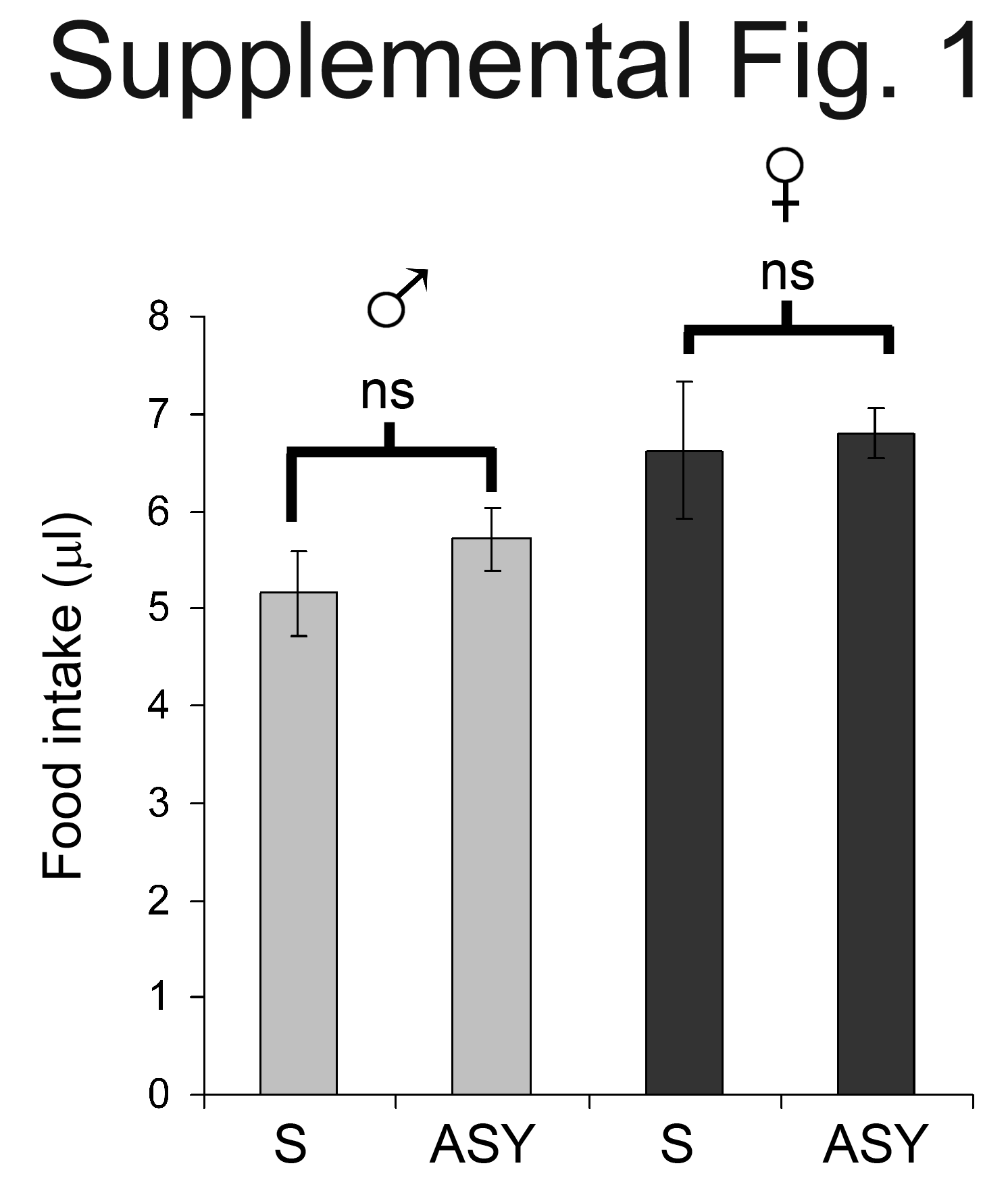

Supplement: Figure S1 — Dietary yeast does not affect food intake. CAFE assays were used to monitor food intake by male and female flies for five days. The provision of 2% yeast extract in the 5% sucrose-water had no effect on food intake. ns = not significant; n = 8–10 flies per diet. (0.08 MB TIF) [file pone.0012062.s001.tif]

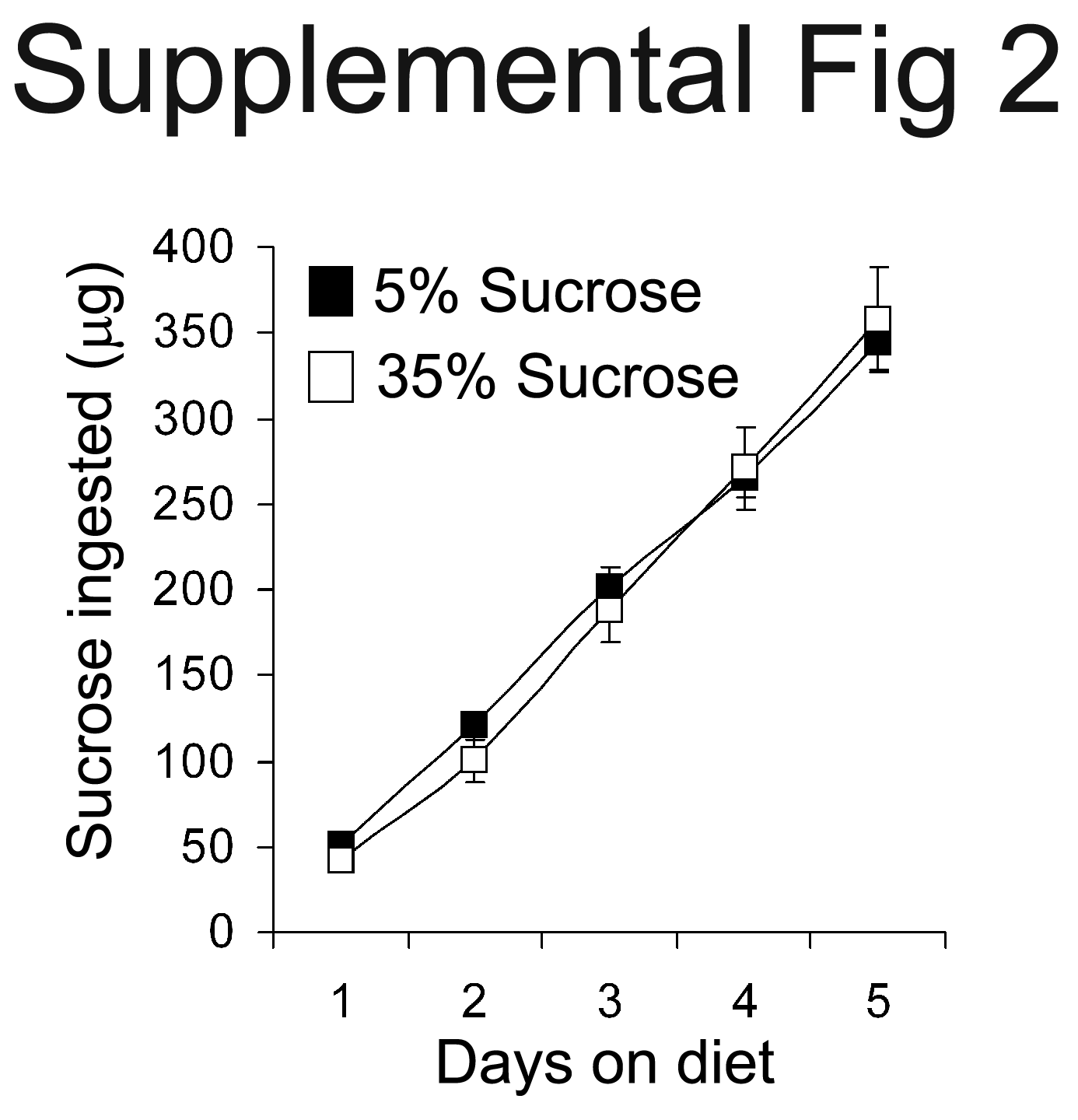

Supplement: Figure S2 — Sucrose ingestion on diets containing different sucrose concentrations. Sucrose ingestion was monitored by CAFE assay for five days. The provision of sucrose at either 5% or 35% in water, had no effect on the total amount of sucrose ingested over a five day period. (n = 8–10 male flies per treatment; P>0.05 by two way ANOVA). (0.08 MB TIF) [file pone.0012062.s002.tif]
